# Chitogel with deferiprone following endoscopic sinus surgery: improved wound healing and microbiome

**DOI:** 10.3389/fsurg.2024.1338209

**Published:** 2024-04-04

**Authors:** Anna Megow, George Bouras, Yazeed Alsuliman, Clare Cooksley, Erich Vyskocil, William Murphy, Sarah Vreugde, Peter-John Wormald

**Affiliations:** Department of Surgery—Otolaryngology, Head and Neck Surgery, University of Adelaide, Adelaide, SA, Australia

**Keywords:** endoscopic sinus surgery, chronic rhinosinusitis (CRS), wound healing, microbiome, chitosan–dextran gel, deferiprone

## Abstract

**Background:**

Adhesion formation, sinus ostial narrowing, and presence of pathogenic bacteria are associated with poor outcomes following endoscopic sinus surgery (ESS) for chronic rhinosinusitis. Chitogel has been shown to improve wound healing, restore a healthier microbiome, and reduce post-operative infections post ESS. Deferiprone has antibacterial properties and has been shown to reduce adhesion formation. The aim of the study was to assess whether the addition of low concentration deferiprone to Chitogel further improves surgical outcomes following ESS compared with Chitogel alone.

**Methods:**

In this double-blinded trial, 45 patients undergoing ESS were prospectively recruited. At the end of the surgery, patients were randomised to receive Chitogel alone, Chitogel with 1 mM of deferiprone, or Chitogel with 5 mM of deferiprone to one side of the sinuses (allowing the other side to serve as control). Patients underwent routine follow-ups with symptom questionnaires and nasoendoscopies performed at 2, 6, and 12 weeks post-operatively. Sinus ostial measurements, microbiology, and microbiome swabs from bilateral middle meatuses were collected intraoperatively and at 12 weeks post-operatively.

**Results:**

A significant improvement in the endoscopic appearance of the sinuses and frontal ostial patency was noted at 12 weeks post-operatively (*p* < 0.05) in all three treatment groups compared with the control. There was no significant difference noted between patients who received Chitogel alone and those who received Chitogel with 1 or 5 mM deferiprone.

**Conclusion:**

Chitogel alone, Chitogel with 1 mM deferiprone, and Chitogel with 5 mM deferiprone used following ESS led to a significant improvement in endoscopic appearance of the sinuses and frontal ostial preservation at 12 weeks post-operatively. No significant difference was found with the addition of deferiprone to Chitogel.

## Introduction

Endoscopic sinus surgery (ESS) is recommended for patients with chronic rhinosinusitis (CRS) resistant to appropriate medical therapy (1). The most common causes for failure of ESS include presence of residual air cells, adhesions, and sinus ostial stenosis (2). Adhesions have been found in up to 56% of cases requiring revision (2). The frontal and sinus ostium have been shown to typically narrow by 3 months post-operatively compared with the baseline with the mean frontal sinus ostial area decreasing by 39.8% and the sphenoid by 42.2% (3). The narrowing of the sinus ostium leads to poor drainage of mucus, infection, and poor access for delivery of topical medication. The bacterial population within the sinonasal environment also influences post-surgical outcomes. The presence of *Staphylococcus aureus* biofilms at the time of ESS worsens post-surgical outcomes, leading to a persistence of sinonasal symptoms, worsening appearance of the sinuses with significant inflammation, and recurrent infection (4–6). In addition, a decrease in beneficial bacteria, *Corynebacterium* and *Cutibacterium*, in the sinonasal microbiome following ESS is associated with an increase in post-operative infections and poorer surgical outcomes (7).

Chitogel, a dissolvable hydrogel dressing composed of chitosan, dextran, and glycerol, can be applied throughout the sinonasal cavity and has been shown to improve wound healing and outcomes following ESS. Chitogel has been shown to improve the endoscopic appearance of the sinuses, reduce ostial stenosis, and reduce post-operative infections (7, 8). A significant increase in beneficial *Corynebacterium* following ESS has been demonstrated following the use of Chitogel (7). Chitogel can further be used to carry topical medication for delivery throughout the sinonasal mucosa following surgery (9).

The iron chelating drug, deferiprone, used for patients with iron overload in β-thalassaemia (10), has both antibacterial and wound healing benefits. Deferiprone disrupts bacterial iron metabolism by chelating the iron in the local bacterial environment and depriving bacteria of this nutrient (11). An *in vitro* study by Richter et al. (11) showed that *Staphylococcus aureus* biofilms commonly found in recalcitrant CRS were susceptible to deferiprone, with significant killing of biofilms noted. Deferiprone has also been shown to have anti-inflammatory properties and to reduce adhesion formation owing to its ability to delay fibroblast migration (12).

When Chitogel is used as a carrier for deferiprone, the latter has been shown to be completely released from Chitogel into the local environment between 48 and 72 h following application (13). The combination of Chitogel with deferiprone following ESS has been investigated by Vediappan et al. (14); Chitogel with 20 mM deferiprone was found to be inferior to Chitogel alone. However, in an animal study assessing Chitogel with lower doses of deferiprone, 1 and 5 mM, a significant reduction in adhesion formation was found compared with Chitogel alone (15). Therefore, further studies to determine whether lower doses of deferiprone with Chitogel result in improved outcomes following ESS are warranted. The effects that deferiprone has on infection rates and microbiomes following ESS are also yet to be explored.

The aim of this study was to determine the effectiveness and safety of deferiprone added to Chitogel at concentrations of 1 and 5 mM compared with Chitogel alone in improving outcomes following ESS. Outcomes assessed include safety, symptom scores, endoscopic appearance, sinus ostial area preservation, infection rate, and microbiome changes.

## Materials and methods

### Study design and participants

This was a double-blinded control study conducted between October 2019 and November 2021. Altogether 45 patients with CRS undergoing primary bilateral full-house functional endoscopic sinus surgery (FH-FESS) were prospectively recruited to receive either Chitogel only, Chitogel with 1 mM deferiprone, or Chitogel with 5 mM deferiprone to one side of the sinuses and nothing to the other side (control) at the end of surgery. The follow-up of patients was performed at 2, 6, and 12 weeks post-operatively. This clinical trial was approved by a tertiary teaching hospital Human Research Ethics Committee (HREC) in South Australia (Reference number HREC/17/TQEH/245. ACTRN12618000577213). Patients over 18 years and able to give informed consent were included in the study. Patients with a shellfish or deferiprone allergy, with a history of hepatitis or blood disorder, or who were pregnant or breastfeeding were excluded from the trial.

### Outcomes

To assess and compare the effectiveness of Chitogel with and without either 1 or 5 mM of deferiprone following surgery, both subjective and objective measures were assessed. The primary outcome for which sample size was calculated was post-operative sinus ostial healing and maintenance of ostial area at 12 weeks post-operatively. Secondary outcomes included: visual analogue scale (VAS) score of sinonasal symptoms, endoscopic appearance scores, rate of post-operative infection, microbiome data, and safety.

### Statistical power analysis

A significant difference in frontal sinus ostia was the primary outcome used for calculation of sample size. The average diameter of the frontal sinus neo-ostia following surgery is 3.5 mm (range 0–11 mm) (16), therefore we chose a significant difference of 3 mm^2^ area in the frontal sinus ostia at 12 weeks with a standard deviation based on ½ the magnitude of the mean difference (i.e., 1.5 mm). Power calculations were performed based on effects assessed at 5% alpha level with 80% statistical power. A sample size was determined to be at least 10 patients in the Chitogel with 1 mM deferiprone group and 10 patients in the Chitogel with 5 mM deferiprone group, thus 20 patients were required in the Chitogel only group for comparison.

### Collection of pre-operative data

Written informed consent was collected from all patients prior to surgery. Furthermore, prior to surgery patients completed a VAS (17) to assess the severity of sinonasal symptoms on each side of the sinonasal tract. Pre-operative computerised tomography (CT) scans of the sinuses were graded according to the Lund–Mackay score (18). Patients were classified into the chronic rhinosinusitis with nasal polyps (CRSwNP) group and the chronic rhinosinusitis without nasal polyps (CRSsNP) group according to the most recent European Position Paper on Rhinosinusitis and Nasal Polyps (EPOS) at the time of the study design (19). Demographic information (including age and gender) and past medical history of asthma, gastroesophageal reflux disease, diabetes mellitus, and smoking status were collected from patients.

### Surgery and collection of intraoperative data

Surgery was performed by one of two operating surgeons and all patients underwent primary bilateral FH-FESS with cold steel and powered instruments with particular care given to preserving mucosa. The patients underwent EFSS grade 1–3 frontal clearance (20). Septoplasty was performed concurrently as required for access to the middle meatus (plication suture was performed to the septal mucosa and incision approximated with this suture, no splits were used in any patient). No patient underwent an extended approach such as a frontal drill-out or medial maxillectomy. At the beginning of surgery, the surgeon collected microbiology and microbiome swabs from the middle meatus on each side of the sinonasal tract under endoscopic guidance. Scoring of the baseline endoscopic appearance of the sinuses on either side was performed prior to randomisation, to assess for adhesions, evidence of infection, oedema, crusting, and granulation tissue. The frontal, maxillary, and sphenoid sinus ostial areas following surgery were determined by measuring height and width of the sinus ostia with a standardised 5 mm measuring probe (see Supplementary Material).

### Deferiprone preparation

Deferiprone [3-hydroxy-1,2-dimethylpyridin-4(1H)-one] was sourced from Sigma-Aldrich (St Louis, MO, United States) and used at a final concentration of 1 or 5 mM. Under sterile conditions, deferiprone was dissolved in 0.3% sodium hydrogen phosphate buffer and 40% glycerol solutions. Once dissolved, the solution was sterilised through a 0.2 µM syringe filter. Sterile deferiprone stock solutions were stored protected from light, at room temperature, and used within 4 weeks.

### Randomisation and intervention

To determine treatment side, small block randomisation was performed using the GraphPad Quickcalcs software (GraphPad Software, San Diego, CA, United States). The surgeon was informed at the end of surgery as to which side had been randomised to receive Chitogel with or without deferiprone, thereby preventing any surgeon-based bias between sides during surgery. The other side of the sinuses received nothing and served as a control. Therefore, the only difference between the operated sides was presence or absence of Chitogel with or without either 1 or 5 mM of deferiprone.

Chitogel was supplied by Chitogel Pty Ltd. (Wellington, New Zealand). Under endoscopic guidance, a malleable cannula was used to apply up to 20 ml of Chitogel with or without deferiprone to fill the floor of the frontal sinus, frontal ostium, frontoethmoidal recess, ethmoid cavity, and sphenoid and maxillary sinuses. To support the middle turbinate, Chitogel was applied to the middle meatus. The nasal tract was left empty to ensure an unobstructed nasal airway, which further ensured patient blinding to treatment side.

### Post-operative care and follow-up

**Figure 1:**
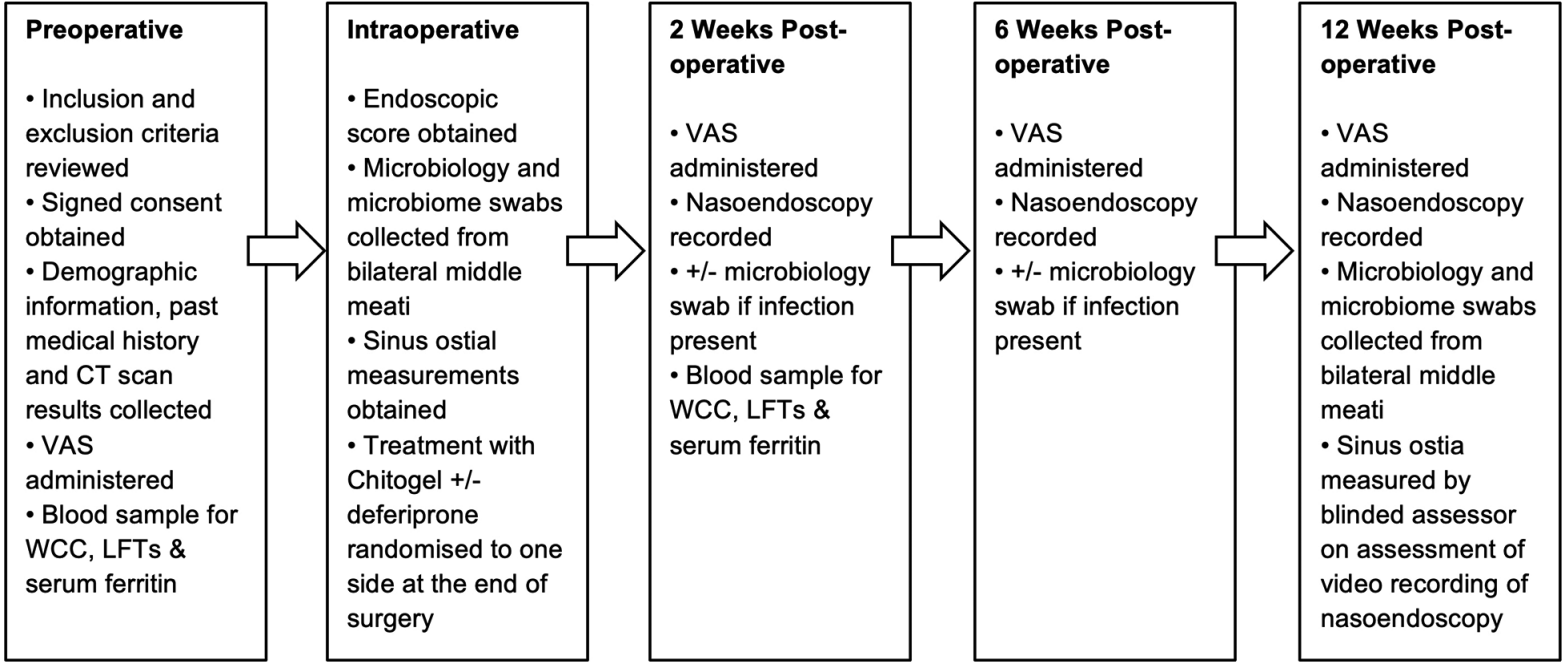
Flow diagram detailing study protocol. WCC, white cell count; LFTs, liver function tests.

Standard follow-up was performed at 2, 6, and 12 weeks post-operatively. A summary of the study protocol at each time point is shown in Figure 1.

All patients received a course of oral antibiotics (amoxicillin/clavulanic acid 875/125 mg twice daily for 7 days) following surgery. In addition, patients with nasal polyps had a 3-week tapering course of prednisolone (25 mg daily for 7 days, 12.5 mg daily for the next 7 days, and 12.5 mg on alternate days for the final 7 days). Starting the day after surgery, patients performed 240 ml saline nasal douches bilaterally four times a day. During the first post-operative visit at 2 weeks, minimal or no Chitogel with or without deferiprone remained within the sinus cavities. Endoscopic debridement of the sinuses was performed on both left and right sides at 2 weeks post-operatively. The debridement was the same for both treated and control sides. It was performed thereafter only if infection was present. Following the 2-week post-operative visit, patients commenced topical steroid, budesonide 1 mg/2 ml (Pulmicort Respules 1 mg/2 ml), which was added to one of the daily saline nasal douches. The infection was managed per standard care with a microbiology swab and culture-directed antibiotics.

### VAS symptom score collection

Patients completed additional VAS questionnaires to assess the severity of sinonasal symptoms on each side of the sinonasal cavity compared with the pre-operative baseline, at the 2-, 6-, and 12-week post-operative visits. Commonly used rhinological VAS questionnaires (21) were adapted for this trial to assess for any differences in symptoms between Chitogel-treated and control sides of the nose. The patients were asked to score symptoms of facial pain or discomfort, bleeding, nasal obstruction, nasal secretions, post-nasal drip, and sense of smell on a scale of 1–10 (where 0 indicated absence of symptom and 10, severe symptom). A total VAS score for each side of the sinonasal tract was obtained at pre-operative baseline, 2, 6, and 12 weeks post-operatively. As patients had both a treated and control side, quality of life questionnaires that included general symptom questions were not used.

### Endoscopic appearance data collection

Video recordings of nasoendoscopies performed at 2, 6, and 12 weeks post-operatively were reviewed by a surgeon not involved in the care of the patient for blinded assessment. The videos were given in random order to the blinded assessor who scored each side of the sinonasal tract for adhesions, evidence of infection, oedema, crusting, and granulation tissue. A total endoscopic score was obtained for each side of the sinonasal tracts at 2, 6, 12 weeks post-operatively to allow for comparison with baseline intraoperative scores prior to randomisation of treatment.

### Post-operative assessment of sinus ostia

The frontal, maxillary, and sphenoidal sinus ostia were remeasured at 12 weeks post-operatively using a standardised 5 mm measuring probe under endoscopic guidance to measure height and width of the ostia. The final area was determined by a blinded assessor viewing a video recording of the measurements being performed during nasoendoscopy. Ostial area maintained at 12 weeks compared with intraoperative baseline for each sinus (frontal, maxillary, sphenoid) was calculated as a percentage and compared between control and treated sides. If the sinus ostial area measured at 12 weeks was larger than the intraoperative baseline area, then the percentage of ostial area maintained was determined to be 100% as it was evident no post-operative ostial stenosis had occurred.

### Safety monitoring

To assess the safety of topical deferiprone, blood samples for white cell count, liver enzymes, and serum ferritin were collected pre-operatively and at 2 weeks post-operatively. Samples were processed at a commercial diagnostic laboratory (Clinpath Pathology, Adelaide, Australia). Adverse event reporting was also used to determine safety.

### Microbiological samples

Intraoperatively and at 12 weeks post-operatively, a swab (Sigma Transwab®, MWE Medical Wire, Corsham, United Kingdom) was collected from each side of the middle meatus under endoscopic guidance. If patients presented with infection at other time points during the study period, additional swabs were collected per standard care. The samples were sent for microscopy, culture, and sensitivity to a commercial diagnostic laboratory (Clinpath Pathology, Adelaide, Australia). The growth of bacteria on culture was quantified as “scant,” “light,” “moderate,” or “heavy.”

### Definition of infection

For the purposes of this study, we considered that infection was present when there was both an endoscopic score of at least mild mucopurulent discharge on review of the video recording of the nasoendoscopy (as assessed by a blinded reviewer) together with at least “moderate” or “heavy” growth of pathogenic bacteria on the microbiology swab of the middle meatus as determined by a diagnostic laboratory (Clinpath Pathology, Adelaide, Australia). To ensure objectivity, we did not include symptom data for the purposes of defining infection in this study.

### Microbiome collection and DNA extraction

Microbiome samples were collected intraoperatively and at 12 weeks post-operatively. Under endoscopic guidance, a guarded Copan Flocked swab (Copan, Brescia, Italy) was used to collect a microbiome sample from the middle meatus on each side. The microbiome samples were collected from other areas of the sinonasal tract. The guarded swab prevented accidental contamination of the swab on the way to and from the middle meatus. Swab tips were stored in individual sterile cryotubes and transported on ice for storage at −80°C.

DNA was extracted from swabs following manufacturer's instructions using The Qiagen DNeasy Blood and Tissue Kit (Qiagen, Hilden, Germany).

### Microbiome sequencing and analysis

The extracted DNA from the microbiome samples underwent sequencing at the Australian Genome Research Facility (AGRF) (Westmead, Australia). Libraries were generated by amplifying the 341F primer against the V3-V4 hypervariable region of the 16S rRNA gene (CCTAYGGGRBGCASCAG forward primer; GGACTACNNGGGTATCTAAT reverse primer). Sequencing was performed using the Illumina MiSeq platform (Illumina Inc., San Diego, CA, United States).

### Bioinformatics

QIIME2 version 2021.11 (22) was used to process the paired-end fastq files. First, the sequences were denoised and Amplicon Sequence Variants (ASVs) were formed using dada2 with the QIIME 2 plugin q2-dada2 (23). The assigning of taxonomy was conducted against the Silva reference database (99% clustered similarity sequences) using a pre-trained Naïve Bayes classifier as part of the q2-feature-classifier plugin (24). The SATé-enabled phylogenetic placement (SEPP) technique was then used for insertion of the ASVs into the high-quality tree generated from the Silva database (25). A rarefaction cut-off of 100 was chosen as quality control for downstream diversity and taxonomic relative abundance analysis. Furthermore, only patients with all four samples (Chitogel with and without deferiprone-treated and control sinuses, at baseline and 12-week time points) satisfying this cut-off were retained for downstream statistical analysis. In total, the microbiomes of 20 Chitogel only, 8 Chitogel with 1 mM deferiprone, and 9 Chitogel with 5 mM deferiprone patients were retained (148 samples). Taxa were aggregated and compared between samples at the genus level. A relative abundance threshold of 3% and a prevalence threshold of 5% were chosen, as pre-filtering improves the performance of differential abundance detection (26). All relative abundances of genera below these thresholds were aggregated into the catch-all “Other” genus for each sample. Faith's phylogenetic diversity index (27) was used as a measure of alpha diversity and was calculated using the qiime2-diversity plugin with a sampling depth of 500.

### Statistical analysis

Differences in baseline characteristics of clinical trial patients in each treatment arm were compared using Fisher's exact test for age and the Lund–Mackay score; analysis of variance (ANOVA) was used for all other patient characteristics as presented in Table 1. VAS symptom and endoscopic score data were analysed using an additive mixed effects linear model implemented with the lme4 R package, where the patient was coded as a random effect (28). This tests for differences between treatment groups and also between treated and untreated sinuses over time, coding the patient as a random effect. The exact model was *Side* * *Time* *+* *treatment* *+* *(1|Patient),* where Side was defined to be whether the sinus was treated or untreated, Time was the time point, and the treatment was Chitogel only, Chitogel with 5 mM deferiprone, or Chitogel with 5 mM deferiprone. Fitting an interaction term between the Side and Time was done to determine whether there was a significant difference between treated and untreated sinuses at any specific time point. A Box-Cox transformation was applied to the data before running the models where appropriate.

**Table 1:** Baseline characteristics of clinical trial patients in each treatment arm.

Patient characteristics	Chitogel only	Chitogel + deferiprone 1 mM	Chitogel + deferiprone 5 mM	*p*-value
Age, median years (range)	55 (22–75)	58.5 (38–72)	49 (24–68)	0.20
Female, no. (%)	12 (48%)	1 (10%)	3 (30%)	0.09
Male, no. (%)	13 (52%)	9 (90%)	7 (70%)	0.09
CRSwNP, no. (%)	12 (48%)	3 (30%)	9 (90%)	0.02
CRSsNP, no. (%)	13 (52%)	7 (70%)	1 (10%)	0.02
LMS, median (range)	12 (4–22)	8 (3–12)	14 (10–18)	0.01
Asthma, no. (%)	8 (32%)	2 (20%)	1 (10%)	0.43
GORD, no. (%)	8 (32%)	6 (60%)	3 (30%)	0.27
Type 2 diabetes mellitus, no. (%)	1 (4%)	1 (10%)	1 (10%)	0.57
Smoker, no. (%)	1 (4%)	1 (10%)	0 (0%)	0.70
Concurrent septoplasty, no. (%)	19 (76%)	7 (70%)	10 (100%)	0.15

Fisher's exact test was used for age and Lund–Mackay score; ANOVA was used for all other patient characteristics. LMS, Lund–Mackay score; GORD, gastroesophageal reflux disease.

To compare the sinus ostial area difference at 12 weeks post-operatively between treated and control sinuses and also the treatment groups, additive mixed linear effects modelling was used, also coding the patient as a random effect, following a Box-Cox transformation. The specific model was *Side* * *Sinus* *+* *treatment* *+* *(1|Patient)*, where Sinus is Maxillary, Frontal, or Sphenoid sinuses. Fitting an interaction term between Side and Sinus was done to determine whether there was a significant difference between treated and untreated sinuses in each specific sinus type.

Differences in the number of infections between the Chitogel with or without deferiprone-treated and control sides were assessed with a Fisher's exact test (*p* < 0.05).

For microbiome data, downstream statistical analysis was conducted using R v 4.2.0 (29). Differential relative abundance testing for each genus between baseline intraoperative and 12-week time points and treatment groups was tested using ANCOM-BC (30, 31). All *p*-values were adjusted to ensure false discovery rate of 5%.

Differences in Faith's Phylogenetic Diversity between time points, and treatment groups were tested using mixed linear effects models implemented in the lme4 R package, where the patient was coded as a random effect (28).

## Results

### Patient cohort

In total, 45 patients were recruited for this study. From those, 25 patients were recruited into the Chitogel only arm, 10 patients into the Chitogel with 1 mM deferiprone arm, and 10 patients in the Chitogel with 5 mM deferiprone arm. There was heterogeneity in the baseline characteristics of the groups. The median age was 56 years (range from 22 to 75 years), with the group consisting of 29 men (64%) and 16 women (36%). Furthermore, 24 patients (53%) had CRSwNP and 21 patients (47%) had CRSsNP. Pre-operative CT sinus scans showed a median Lund–Mackay score (18) of 12 (range 3–22). Approximately 17 patients (38%) had gastroesophageal reflux disease, 11 (24%) had asthma, 3 (7%) had type 2 diabetes mellitus, and there were 2 (4%) smokers. Septoplasty at the time of surgery was performed in 36 (80%) patients. The baseline characteristics of the clinical trial patients in each treatment arm are provided in Table 1.

Altogether 42 patients completed follow-up at the time points 2, 6, and 12 weeks post-operatively. Three patients that failed to attend follow-up were all in the Chitogel only group (two failed to attend follow-up at 12 weeks and one at 6 weeks post-operatively); data collected from the follow-up time points attended by these patients were included in the analysis.

Of the 25 patients that received Chitogel only, 13 (52%) were randomised to receive Chitogel on the left and 12 (48%) on the right. In both the Chitogel with 1 mM deferiprone and Chitogel with 5 mM deferiprone groups, the patients were randomised to receive treatment on the left and right sides equally.

### VAS symptom scores

All patients had an improvement in total VAS scores following surgery compared with the pre-operative baseline. All three treatments showed a trend of decreasing scores after 6 and 12 weeks post-operatively. When comparing total VAS scores between the treated and untreated sides, a trend for improved scores on the treated side was noted across all post-operative time points in all the three treatment groups, but the difference was not statistically significant (*p* = 0.32 at the 12-week post-operative time point). There was also no significant difference between the three treatments (*p* = 0.29 and *p* = 0.25 comparing Chitogel with 1 mM deferiprone and Chitogel with 5 mM deferiprone vs. Chitogel only, respectively).

### Endoscopic appearance

**Figure 2:**
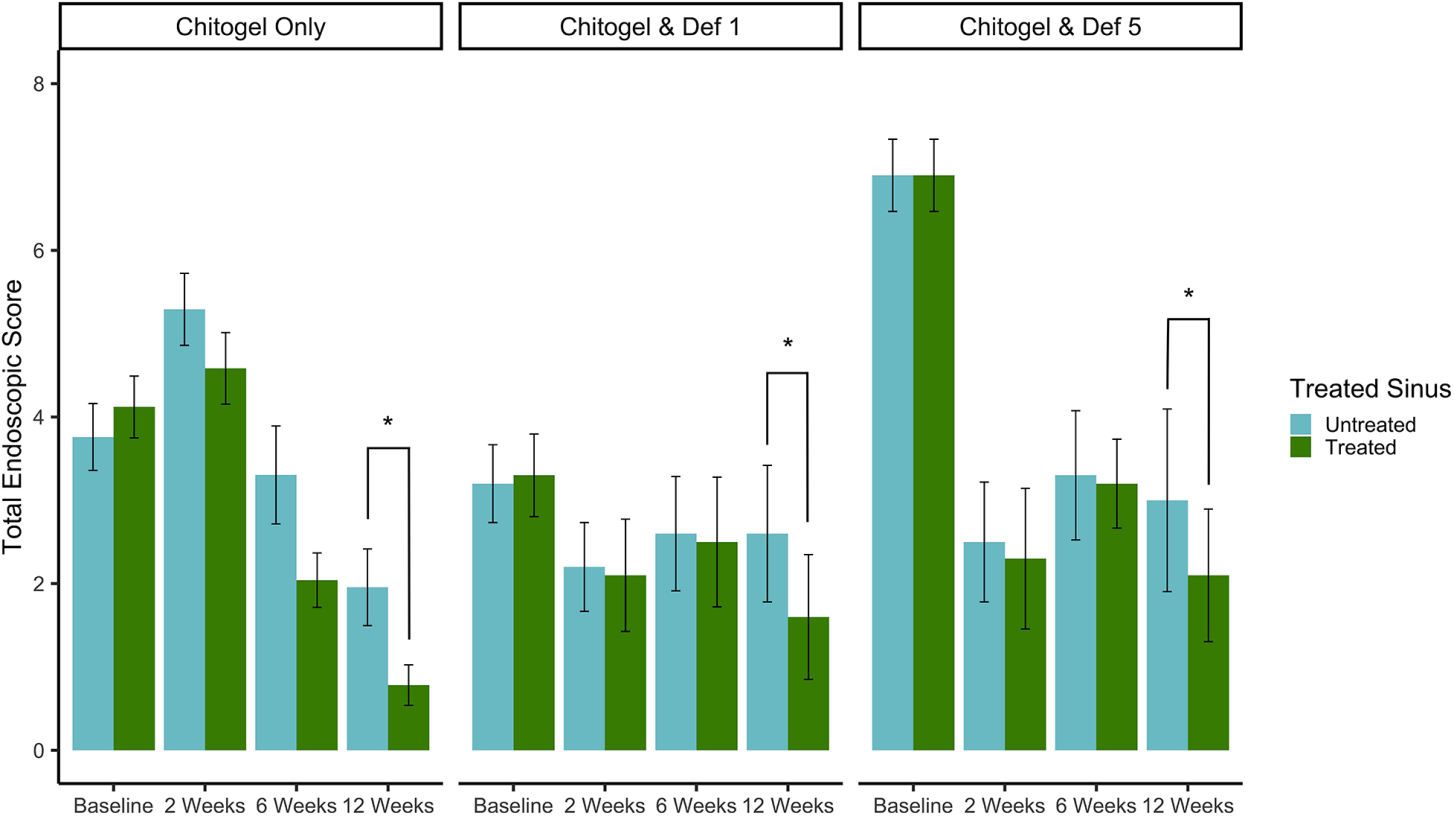
Total endoscopic scores at intraoperative baseline, 2, 6, and 12 weeks post-operatively for control and treated sides in the Chitogel only group, Chitogel with 1 mM deferiprone group, and Chitogel with 5 mM deferiprone group. Data represent the mean ± SEM. *p = 0.03, additive mixed effects linear model. SEM, standard error of the mean; Def 1, 1 mM deferiprone; Def 5, 5 mM deferiprone.

All patients had an improvement in endoscopic appearance of the sinuses following surgery compared with the pre-operative baseline. All three treatments showed decreasing scores after 6 and 12 weeks post-operatively. At 12 weeks, there was a statistically significant difference of improved scores on the treated side compared with the untreated side (*p* = 0.03, estimated coefficient of 0.91-point reduction in the treated sinus vs. untreated at 12 weeks post-operatively) across the three groups. There were no significant differences between the three treatments (*p* = 0.38 and *p* = 0.37 comparing Chitogel with 1 mM deferiprone and Chitogel with 5 mM deferiprone vs. Chitogel only, respectively), suggesting the difference between treated and untreated sinus at 12 weeks post-operatively was present in all the three groups see Figure 2.

### Ostial measurements

**Figure 3:**
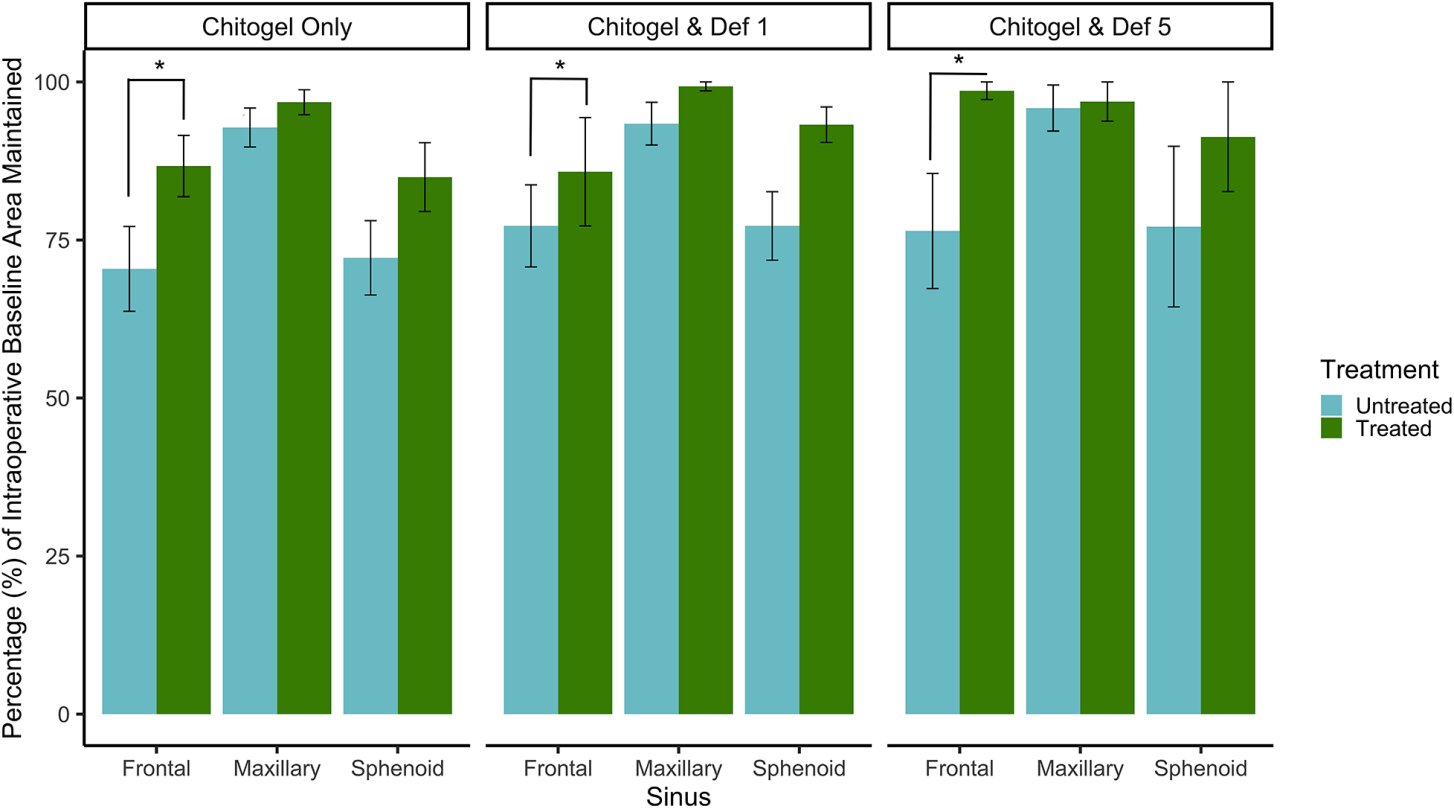
Percentage (%) of intraoperative baseline ostial area maintained for the frontal, maxillary, and sphenoid sinuses at 12 weeks post-operatively for control and treated sides in the Chitogel only group, Chitogel with 1 mM deferiprone group, and Chitogel with 5 mM deferiprone group. Data represent the mean ± SEM. *p = 0.04, additive mixed effects linear model. SEM, standard error of the mean; Def 1, 1 mM deferiprone; Def 5, 5 mM deferiprone.

The baseline ostial area (intraoperatively measured) was compared with the ostial area measured by a blinded assessor 12 weeks post-operatively. The percentage of the baseline area maintained after 12 weeks post-operatively was compared between untreated and treated sinuses in all three treatment groups, for all three sinuses. All three sinuses showed an increased proportion of baseline area maintained in the treated sinus across the three treatments as can be seen in Figure 3. The difference was statistically significant for the frontal sinus (*p* = 0.04), and showed a strong trend for sphenoid (*p* = 0.09) and maxillary (*p* = 0.26) sinuses. There was no significant difference between the three treatment groups (*p* = 0.52 and *p* = 0.18 comparing Chitogel with 1 mM deferiprone and Chitogel with 5 mM deferiprone vs. Chitogel only, respectively), although the Chitogel with 5 mM deferiprone group showed a trend of higher ostial baseline area maintained after 12 weeks compared with the Chitogel only group.

### Rates of infection

Approximately 20 patients developed infections in the middle meatus during follow-up; 7 patients at 2 weeks, 6 at 6 weeks, and 7 at 12 weeks. *Staphylococcus aureus* was identified by a diagnostic laboratory to be the most common cause of infection accounting for infection in 12 patients; this was followed by *Pseudomonas aeruginosa,* identified in 4 patients, *Klebsiella aerogenes* in 2 patients, *Citrobacter koseri* in 1 patient, and *Serratia liquefaciens* in 1 patient.

In the Chitogel only group, 10 patients developed a post-operative infection; there was a significant decrease in infections in the Chitogel only-treated sinuses (12%) compared with the control sinuses (40%) (*p* = 0.05).

In the Chitogel with 1 mM deferiprone group, six patients developed a post-operative infection; there were no significant differences in infections in the Chitogel with 1 mM deferiprone-treated sinuses (40%) compared with the control sinuses (60%) (*p* = 0.66).

**Figure 4:**
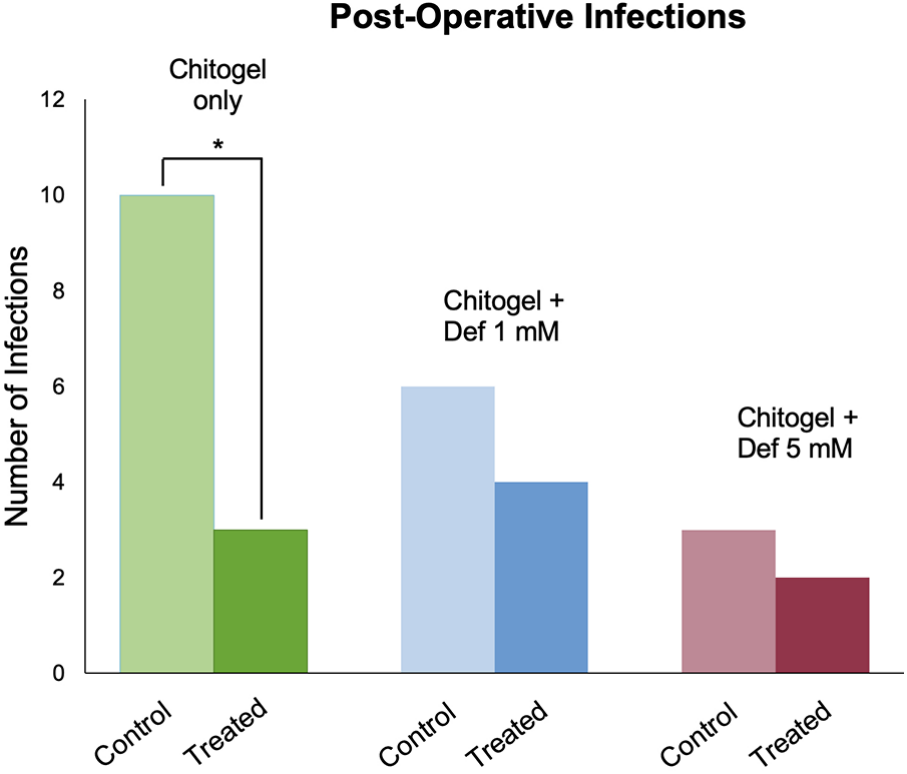
Post-operative infections in the Chitogel only, Chitogel with 1 mM deferiprone, and Chitogel with 5 mM deferiprone groups in the control and treated sides. *p = 0.05, Fisher's exact test. Def, deferiprone.

In the Chitogel with 5 mM deferiprone group, four patients developed a post-operative infection; there were no significant differences in infections in the Chitogel with 5 mM deferiprone-treated sinuses (20%) compared with the control sinuses (30%) (*p* = 1.00) (see Figure 4).

### Microbiome

**Figure 5:**
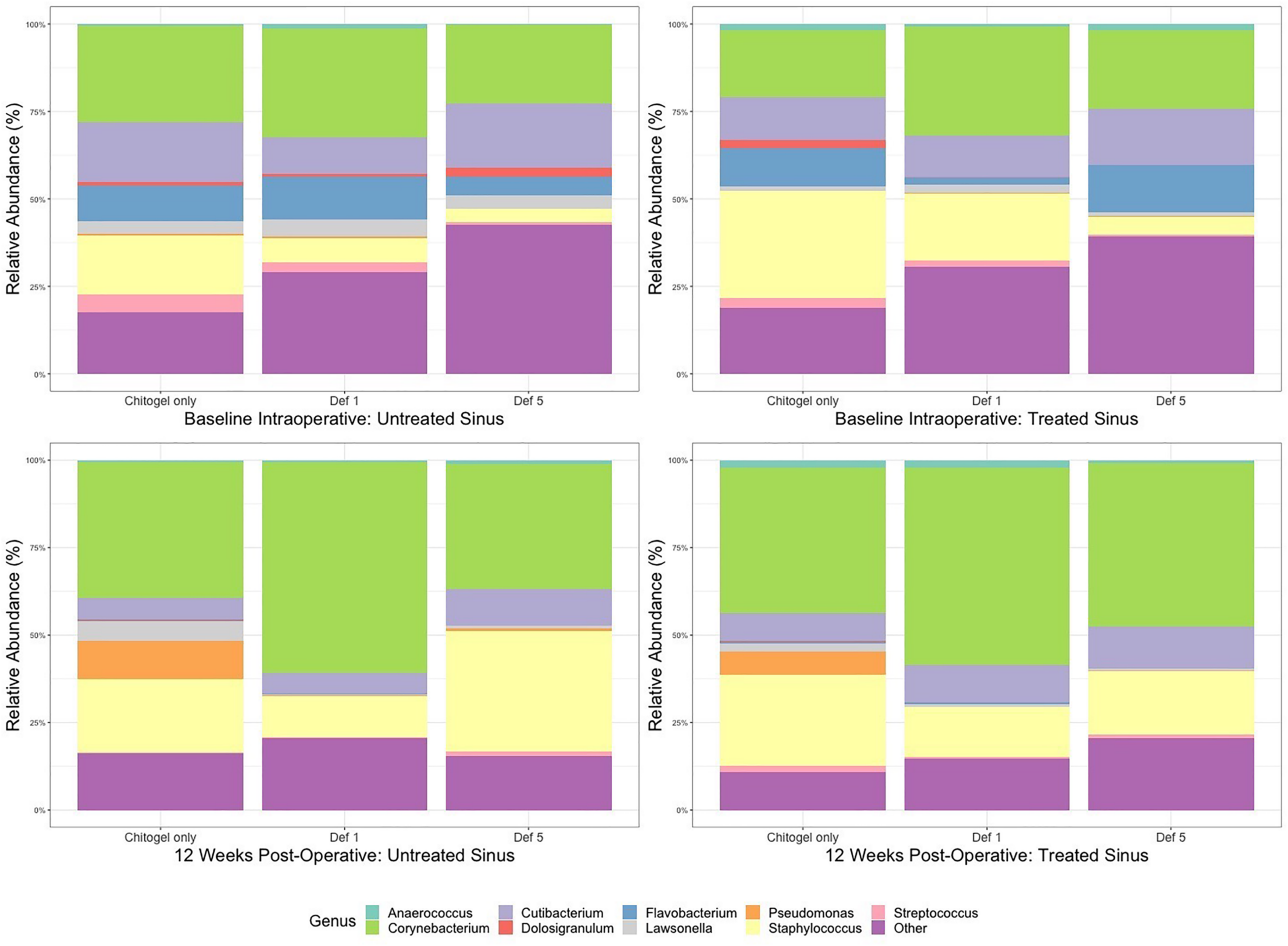
Mean relative abundance (%) of microbiota at intraoperative baseline and 12 weeks post-operatively in the Chitogel only, Chitogel with 1 mM deferiprone, and Chitogel with 5 mM deferiprone groups. Def, deferiprone.

From the middle meatus, nine genera were detected such that they passed thresholds described in the methods: *Anaerococcus, Corynebacterium, Cutibacterium, Dolosigranulum, Flavobacterium, Lawsonella, Pseudomonas, Staphylococcus, Streptococcus*, and the catch-all “Other.” The most abundant genera detected over all three time points were *Corynebacterium*, *Staphylococcus*, and *Cutibacterium* (see Figure 5).

**Figure 6:**
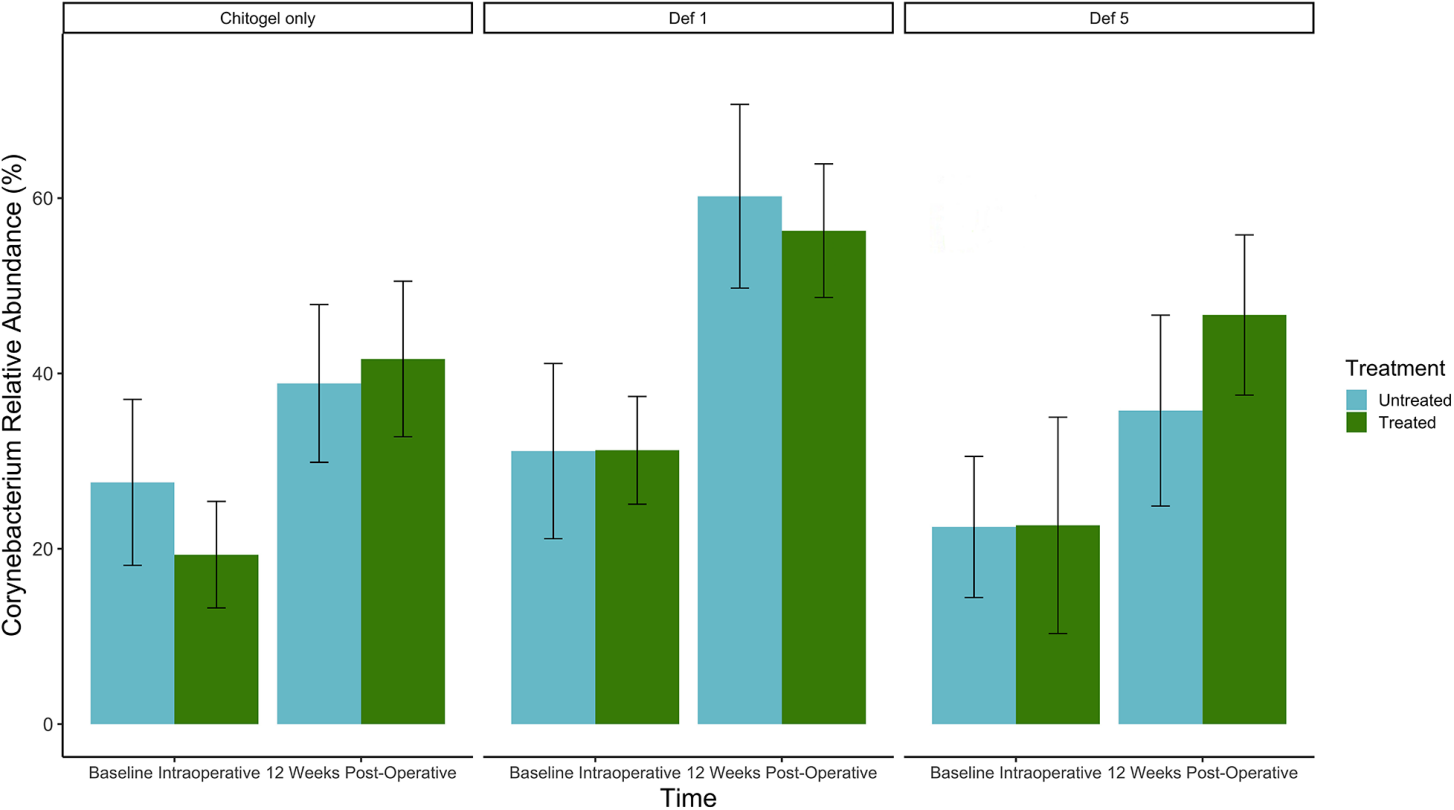
Mean relative abundance (%) of *Corynebacterium* at intraoperative baseline and 12 weeks post-operatively in the Chitogel only, Chitogel with 1 mM deferiprone, and Chitogel with 5 mM deferiprone group. Data represent the mean ± SEM. ANCOM-BC model. SEM, standard error of the mean. Def, deferiprone.

The proportion of *Corynebacterium* increased over time in all sinuses (treated or untreated). The increase was higher in the treated sinuses for the Chitogel only group and the Chitogel with 5 mM deferiprone group, but not the Chitogel with 1 mM deferiprone group. In the Chitogel only group, the relative abundance in the treated side increased from 19.3% at baseline to 41.7% at 12 weeks post-operatively, compared with the untreated side, which was 27.6% at baseline and 38.9% at 12 weeks post-operatively. In the Chitogel with 5 mM deferiprone group, the relative abundance in the treated side increased from 22.7% at baseline to 46.7% at 12 weeks post-operatively, compared with the untreated side, which was 22.5% at baseline and 35.8% at 12 weeks post-operatively. However, this trend was not statistically significant (*p*_adj_ = 0.5) (see Figure 6 and Table 2).

**Table 2:** The mean relative abundance (%) of the three most common genera found in the control and treated arms in the Chitogel only, Chitogel with 1 mM deferiprone, and Chitogel with 5 mM deferiprone groups at baseline and 12 weeks post-operatively.

Genera	Time point	Mean relative abundance (%)					
		Chitogel only		Chitogel with 1 mM deferiprone		Chitogel with 5 mM deferiprone	
		Control	Treated	Control	Treated	Control	Treated
*Corynebacterium*	Baseline	27.58	19.33	31.15	31.23	22.49	22.67
	12 weeks post-operatively	38.86	41.65	60.21	56.29	35.77	46.67
*Staphylococcus*	Baseline	16.83	30.54	6.9	19.26	3.77	5.1
	12 weeks post-operatively	20.85	26.09	11.9	14.1	34.46	18.03
*Cutibacterium*	Baseline	17.16	12.23	10.54	11.83	18.28	15.98
	12 weeks post-operatively	6.14	8.04	5.89	10.92	10.26	11.88

**Figure 7:**
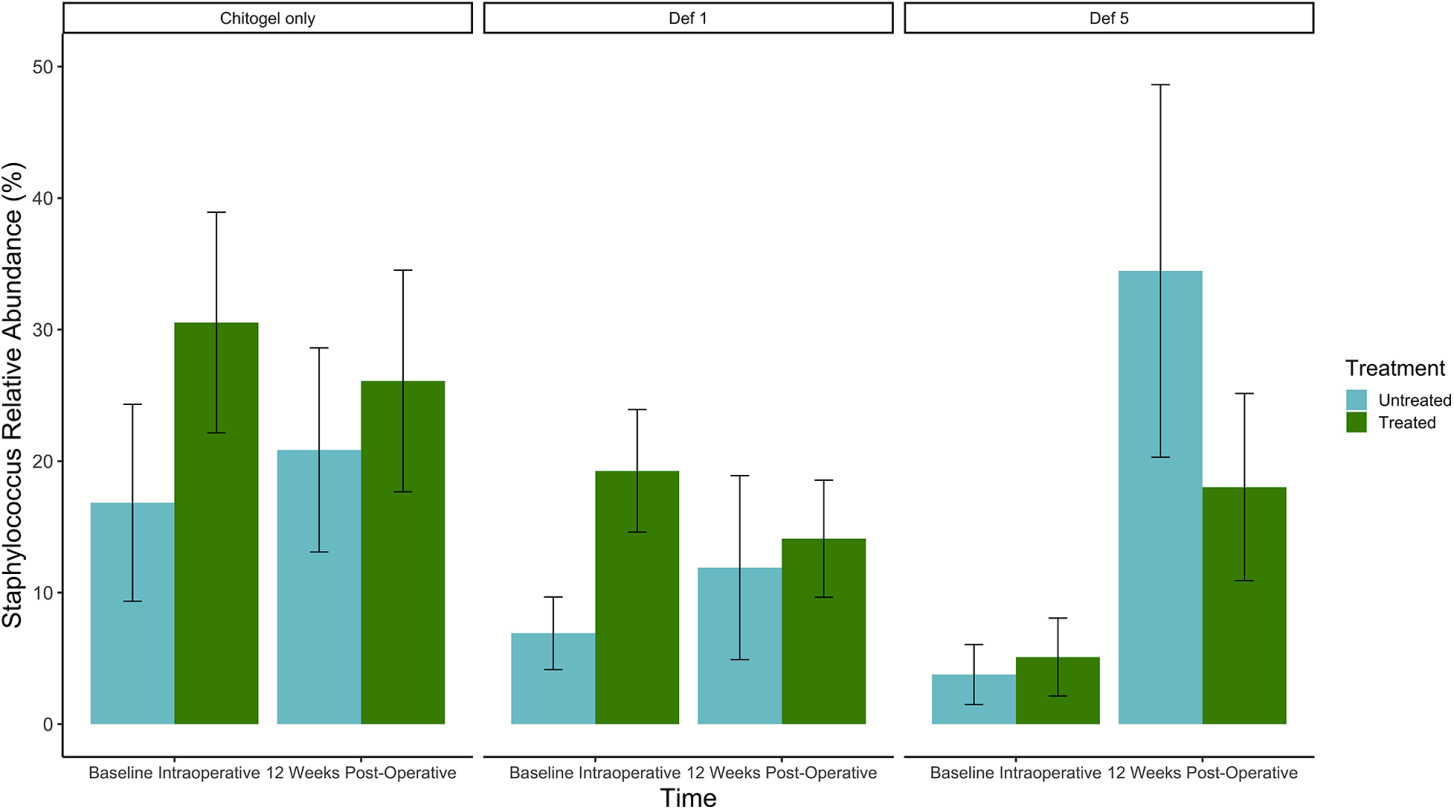
Mean relative abundance (%) of *Staphylococcus* at intraoperative baseline and 12 weeks post-operatively in the Chitogel only, Chitogel with 1 mM deferiprone, and Chitogel with 5 mM deferiprone group. Data represent the mean ± SEM. ANCOM-BC model. SEM, standard error of the mean. Def, deferiprone.

The proportion of *Staphylococcus* increased over time in all three treatments in the untreated sinus, but not the treated sinus. For the Chitogel only and Chitogel with 1 mM deferiprone groups, the mean relative abundance increased from 16.8% to 20.9% and 6.9% to 11.9% between baseline and 12 weeks post-operatively in the untreated sinus, respectively, while the mean relative abundance decreased from 30.5% to 26.1% and 19.3% to 14.1% between baseline and 12 weeks post-operatively in the treated sinuses. This trend was also not statistically significant (*p*_adj_ = 0.15) (see Figure 7 and Table 2).

**Figure 8:**
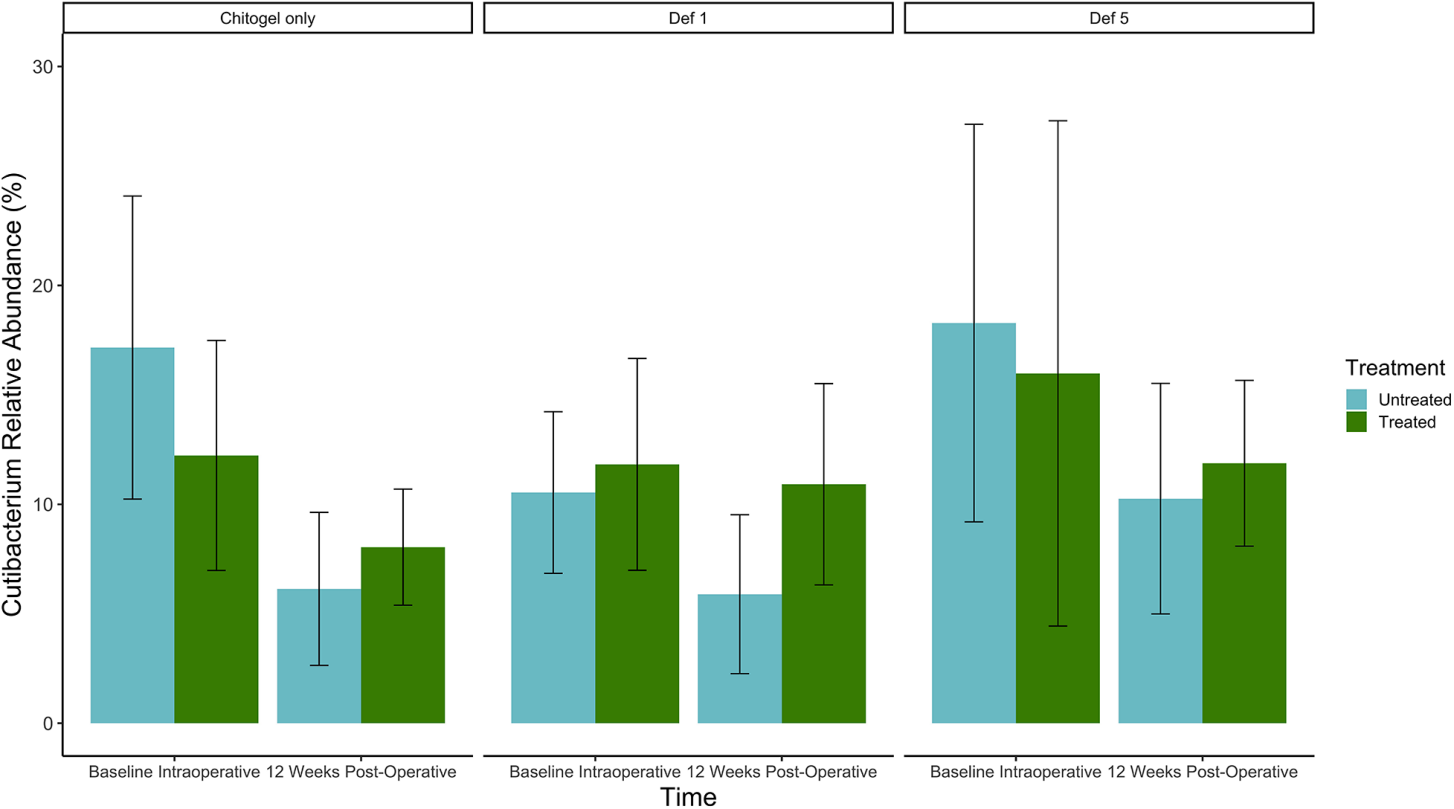
Mean relative abundance (%) of *Cutibacterium* at intraoperative baseline and 12 weeks post-operatively in the Chitogel only, Chitogel with 1 mM deferiprone, and Chitogel with 5 mM deferiprone group. Data represent the mean ± SEM. ANCOM-BC model. SEM, standard error of the mean. Def, deferiprone; mM, millimolar.

The proportion of *Cutibacterium* decreased over time in all three treatments in both sinuses, but the size of the decrease was much higher in the untreated sinuses than the treated. The mean relative abundances decreased from 17.2%, 10.5%, and 18.3% to 6.1%, 5.9%, and 10.3% between baseline and 12 weeks in the untreated sinuses (Chitogel only, Chitogel with 1 mM deferiprone, and Chitogel with 5 mM deferiprone, respectively), while for the treated sinuses, the mean relative abundances decreased from 12.2%, 11.8%, and 16.0% to 8.0%, 10.9%, and 11.9% between baseline and 12 weeks (*p*_adj_ = 0.14) (see Figure 8 and Table 2).

In terms of diversity, Faith's Phylogenetic Diversity was used as a measure. There was an observed trend of increase in Faith's Phylogenetic Diversity after 12 weeks in the treated sinus compared with the untreated sinus in the Chitogel with 1 mM deferiprone and Chitogel with 5 mM deferiprone groups (*p* = 0.2 and *p* = 0.16).

### Safety monitoring

No significant change was noted in white cell count, liver enzymes, or serum ferritin levels at 2 weeks following administration of the topical deferiprone compared with baseline. No adverse outcomes were found following use of Chitogel or deferiprone, both were well tolerated by all patients.

## Discussion

In the hope of achieving optimal wound healing and the best possible outcome following ESS, nasal packing is commonly used by surgeons. In this preliminary, double-blinded control study there appeared to be no significant difference in outcomes between Chitogel alone or Chitogel with 1 or 5 mM deferiprone when used following ESS.

Chitogel alone and with either 1 or 5 mM deferiprone led to a significant increase in the proportion of baseline frontal ostial area maintained at 12 weeks post-operatively and a significant improvement in the endoscopic appearance of the sinuses at 12 weeks post-operatively compared with the control; however, no significant difference between the three treatment groups was evident. Previous studies have shown that Chitogel leads to a significant improvement in endoscopic appearance of the sinuses and reduced ostial stenosis following ESS (7, 8). Therefore, whether the addition of deferiprone made any difference to these outcomes is not evident in this study, but Chitogel with deferiprone at 1 and 5 mM concentrations was not inferior to Chitogel alone. In light of previous studies that have demonstrated deferiprone's anti-inflammatory and anti-adhesion properties (12, 15), the aim of this study was to further improve post-surgical outcomes with the addition of deferiprone, although this study showed no added benefit with the addition of deferiprone to Chitogel. The sample size was small and further study would be warranted.

The sinonasal symptoms were assessed using VAS. To assess symptoms following treatment with Chitogel alone and with either 1 or 5 mM deferiprone compared with control, patients were required to assess symptoms for each side of the nasal cavity separately. Although there was a trend for improvement in VAS in treated sinuses compared with control, a limitation of this study design was that patients found it difficult to differentiate some of the symptoms such as post-nasal drip and smell between the sides. Whether or not a septoplasty was performed would have been a confounding factor in assessing the symptom of nasal obstruction.

This is the first study examining the sinonasal microbiome following topical deferiprone use. Previous *in vitro* work has demonstrated the *Staphylococcus aureus* biofilm killing potential of deferiprone (11). A recent clinical trial examining the effect of Chitogel on the sinonasal microbiome following ESS has shown that Chitogel led to a significant increase in beneficial commensals, *Corynebacterium* and *Cutibacterium* (7). This study has shown a trend for an improvement in microbiome health, with a trend for increase in *Corynebacterium* and *Cutibacterium* in the middle meatus compared with the control and a trend for decreased pathogenic *Staphylococcus* compared with the control. There was no difference noted between groups who received Chitogel alone or Chitogel with 1 or 5 mM deferiprone. In keeping with the trend for improved microbiome, there was a significant reduction in infections in the Chitogel only group. In terms of infections, the addition of 1 or 5 mM of deferiprone to Chitogel did not appear to be as effective as Chitogel alone; there were fewer infections with the addition of deferiprone compared with the control, but this was not significant. The presence of pathogenic bacteria and post-operative infections lead to poorer outcomes following surgery (4, 6). Although deferiprone has been shown to have antibacterial and antibiofilm properties (11, 13), a study with a larger sample size would be warranted to further determine the effect of topical deferiprone in the sinonasal microbiome.

A limitation of the study is the heterogeneity in the treatment groups (e.g., age, gender, presence or absence of nasal polyps, past medical history, requirement for frontal drill out or septoplasty). In the design of this trial, each patient had a treated and control side of the sinonasal tract and by only comparing changes in the treatment side relative to changes in the control side within each patient (i.e., each patient acted as their own internal control), we attempted to account for any differences due to heterogeneity. Despite this, a trial with a larger sample size would be required to minimise the effect of heterogeneity. Furthermore, future study with participants classified per the EPOS 2020 (1) guidelines based on endotypes (rather than CRSsNP and CRSwNP) would allow better comparison by taking into account the heterogeneity in immunologic responses across various CRS endotypes.

Vediappan et al. (14) previously assessed the use of Chitogel with a higher concentration of deferiprone following ESS and found that Chitogel with deferiprone at 20 mM was safe and tolerable; however, it was inferior to Chitogel alone. We have found that Chitogel with deferiprone at lower concentrations of 1 and 5 mM to be as effective as Chitogel alone; however, larger studies are required to further explore whether there is any additional benefit to the use of deferiprone with Chitogel.

## Conclusion

A significant improvement in endoscopic appearance of the sinuses and frontal ostial preservation at 12 weeks following ESS was noted in patients who received Chitogel alone, Chitogel with 1 mM deferiprone, and Chitogel with 5 mM deferiprone. The addition of at deferiprone of 1 and 5 mM did not significantly improve outcomes compared with Chitogel alone.

## Data Availability

The original contributions presented in the study are publicly available. This data can be found here: SRA BioProject, accession number PRJNA1091374 (https://www.ncbi.nlm.nih.gov/bioproject/?term=PRJNA1091374).
