# Supplementary material for: Chitogel with deferiprone following endoscopic sinus surgery: improved wound healing and microbiome
Source: Front Surg. 2024 Apr 4;11:1338209. doi: 10.3389/fsurg.2024.1338209 (PMC11024462; doi:10.3389/fsurg.2024.1338209)
Supplement: Supplementary file 1 [file Datasheet1.pdf]

Supplementary Document – Methodology for Endoscopic Measurement of Sinus Ostia

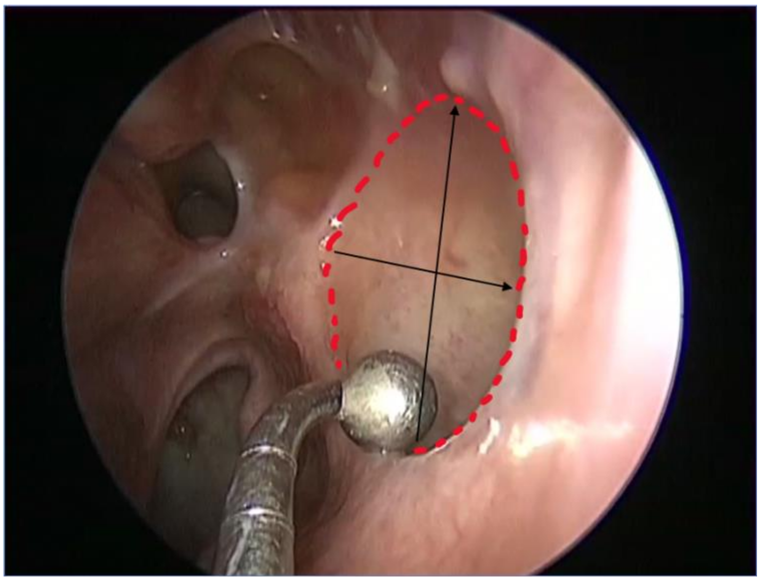

Figure: Endoscopic view of left maxillary sinus ostia measured with standard 5 mm probe at 12 weeks post operative. Broken line representing area of ostium. The black arrows represent the height and width dimensions being measured.

Methodology:

- The frontal, maxillary and sphenoidal sinus ostia were measured immediately post-operatively and at 12 weeks post-operatively.
- Under endoscopic guidance a standardised 5 mm measuring probe was used to measure height and width (or antero-posterior and lateral) dimensions of the ostia. These measurements were used to calculate "ostial area".
- Ostial area maintained at 12 weeks compared to intraoperative baseline for each sinus (frontal, maxillary, sphenoid) was calculated as a percentage and compared between control and treated sides. If the sinus ostial area measured at 12 weeks was larger than intraoperative baseline area, then the percentage of ostial area maintained was determined to be 100% as it was evident no post-operative ostial stenosis had occurred.
